# Beyond duty hours: leveraging large-scale paging data to monitor resident workload

**DOI:** 10.1038/s41746-019-0165-2

**Published:** 2019-09-09

**Authors:** Amit Kaushal, Laurence Katznelson, Robert A. Harrington

**Affiliations:** 1 Department of Medicine, Veterans Affairs Palo Alto Health Care System, Palo Alto, CA 94304 USA; 2 Department of Neurosurgery, Stanford School of Medicine, Stanford University, Stanford, CA 94305 USA; 3 Department of Medicine, Stanford School of Medicine, Stanford University, Stanford, CA 94305 USA

**Keywords:** Education, Data mining

## Abstract

Monitoring and managing resident workload is a cornerstone of policy in graduate medical education, and the duty hours metric is the backbone of current regulations. While the duty hours metric measures hours worked, it does not capture differences in intensity of work completed during those hours, which may independently contribute to fatigue and burnout. Few such metrics exist. Digital data streams generated during the usual course of hospital operations can serve as a novel source of insight into workload intensity by providing high-resolution, minute-by-minute data at the individual level; however, study and use of these data streams for workload monitoring has been limited to date. Paging data is one such data stream. In this work, we analyze over 500,000 pages—two full years of pages in an academic internal medicine residency program—to characterize paging patterns among housestaff. We demonstrate technical feasibility, validity, and utility of paging burden as a metric to provide insight into resident workload beyond duty hours alone, and illustrate a general framework for evaluation and incorporation of novel digital data streams into resident workload monitoring.

## Introduction

Resident workload has been in the public conscience since at least 1984, when the death of college student Libby Zion was proposed to be linked to resident fatigue.^1^ In subsequent years, additional studies have suggested that long work hours are associated with adverse events affecting both house officers and potentially their patients.^2,3^ To limit resident overwork, resident workload restrictions were recommended and ultimately mandated. The primary metric under restriction is hours worked, known as duty hours. Several notable studies have been unable to conclusively demonstrate improvement in outcomes with duty hours regulation.^4–6^

One hypothesis is that it is not just hours worked, but what happens during those hours, that contributes to workload and fatigue. The pager is inextricably intertwined with the daily routine of the medicine house officer. Analysis of large-scale paging data has the potential to provide high resolution, minute-by-minute information about not just the hours worked, but the nature and intensity of work completed during those hours. Most studies to date involve manual analysis of smaller-scale paging datasets^7–10^; no studies have characterized paging patterns in an academic medical residency program across multiple years, and even basic questions such as “How many pages does an internal medicine resident receive per year” remain unanswered.

## Results

### Baseline paging characteristics

To demonstrate the utility of paging data in the discussion around resident workload, we first characterize baseline paging burden and frequency. We next explore whether paging burden is a metric that demonstrates variation even when duty hours are similar. We subsequently investigate whether observed variation is informative—that is, whether increased paging is associated with other metrics known to be related to workload.

In the two-year period of this study, residents of the internal medicine program received a total of 502,293 pages (Table 1). Interns (R1s) received an average of 2924 pages per year, while second-year residents (R2s) received an average of 1898 pages, and third-year residents (R3s) an average of 1492 pages. Over half of all pages were sent to interns, and on inpatient wards and subspecialty services, 57 to 87% of pages were fielded by interns.

**Table 1:** Program-wide paging burden, by year of training and service

Service type	Service	R1 (*n* = 91)	R2 (*n* = 71)	R3 (*n* = 68)	Total	Percentage (%)	Resident-days	Average per resident per day
Inpatient-Wards	Wards (University)	89,740	14,164	17,961	121,865	24.3	7245	16.8
Inpatient-Wards	Wards (VA)	49,618	22,726	16,630	88,974	17.7	7149	12.4
Inpatient-Night Float	Nights (University)	41,731	31,051	24,706	97,488	19.4	3961	24.6
Inpatient-Night Float	Nights (VA)	12,478	15,596	10,998	39,072	7.8	2018	19.4
Inpatient-Subspecialty	Oncology	30,430	2815	1787	35,032	7.0	2497	14.0
Inpatient-Subspecialty	Hematology	14,077	5552	4725	24,354	4.8	1818	13.4
Inpatient-Subspecialty	Cardiology	13,905	4343	2466	20,714	4.1	1754	11.8
Critical care	CCU	4052	19,278	7833	31,163	6.2	2777	11.2
Critical care	ICU (University)		9714	4253	13,967	2.8	2168	6.4
Critical care	ICU (VA)	3051	282	206	3539	0.7	1776	2.0
Other	Consult	2906	4161	4835	11,902	2.4	4103	2.9
Other	Outpatient	2475	2468	2814	7757	1.5	2673	2.9
Other	Elective (nonclinical)		637	834	1471	0.3	706	2.1
Other	Elective (inpatient)		1016	310	1326	0.3	208	6.4
Other	ED	424	285	541	1250	0.2	717	1.7
Other	Misc	1179	701	539	2419	0.5	1066	2.3
	Total	266,066	134,789	101,438	502,293	100	42,636	11.8
	Average, per resident per year	2924	1898	1492	2184			

502,293 pages were analyzed over the two-year period of the study. Interns received 53.0% of all pages during the study period. On average, interns received 2924 pages per year, compared with 1898 for a second-year resident and 1492 for a third-year resident. 85.1 percent of all pages were sent to residents on inpatient wards (42.0%), night float (27.2%), and inpatient subspecialty (15.9%) services

*CCU* coronary care unit, *ICU* intensive care unit, *ED* emergency department

### Patterns of variation

**Fig. 1:**
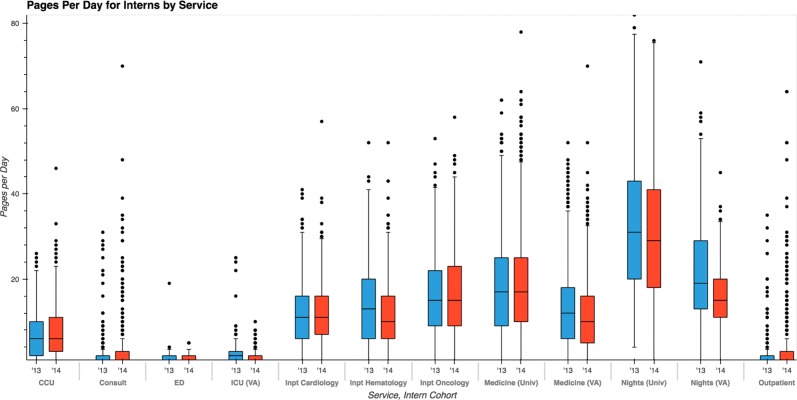
Intern pages received per day, by clinical service and year of study. Different services receive pages at different frequencies; however, within any given service, distribution of pages received per day is relatively consistent year-over-year. In both years under study, the University night float service received the greatest number of pages per day for interns, and also largest interquartile range. (Blue = 2013–14 academic year, red = 2014–15 academic year. Boxplot elements: center line, median; box limits, upper and lower quartiles; whiskers, 1.5× interquartile range; points, outliers)

Pages are not distributed uniformly across hours worked; patterns of variation emerge across services and year of training, and during time of day within a service. The most paged role in the training program is night-float intern covering the University hospital, where interns received a median of 30 pages per night (Fig. 1). Outpatient and elective rotations generated the least number of pages per intern. Trends within a service are consistent year-over-year.

**Fig. 2:**
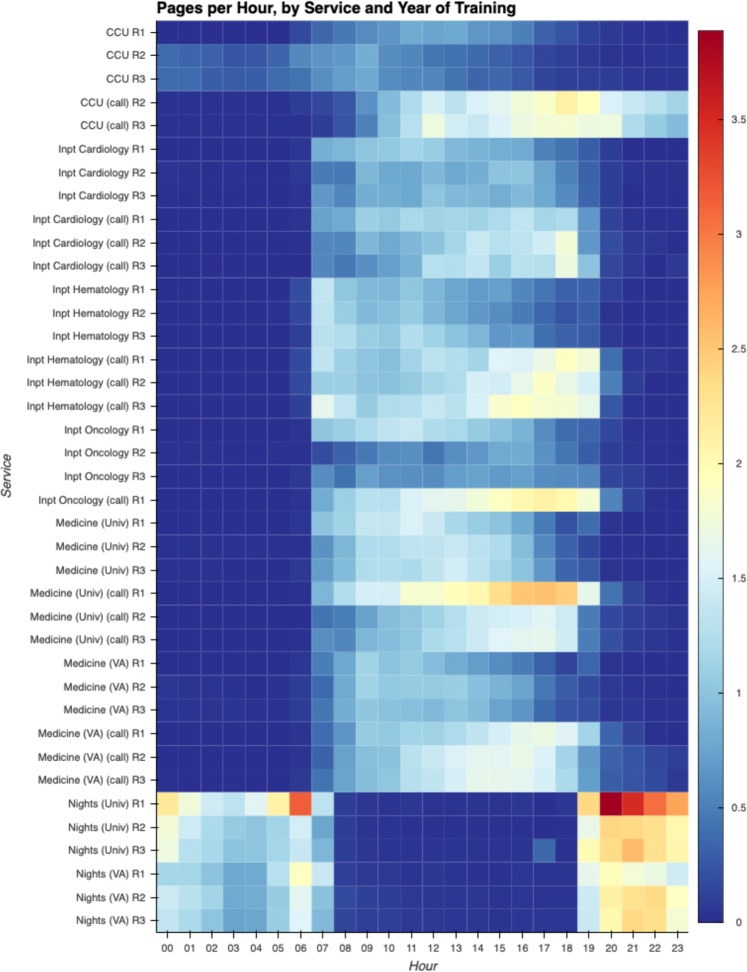
Pages per hour, by service and year of training. Even within a given service, not all hours of the day are equal. A heatmap of paging patterns helps visualize hotspots across the program. The 8 p.m. hour for interns covering the University night float service is, on average, the most frequently paged role and time in the entire residency program. On the night float services at both hospitals interns are paged in a bimodal distribution, with consistently heavy paging from ~8 p.m. to 1 a.m., and then another peak at 6 a.m., anecdotally known to house staff as the “morning labs” pages, when the night float team must respond to new abnormalities discovered on that morning’s labs. For the inpatient medicine and subspecialty services, on non-call days, paging is relatively steady throughout the day. On call days in contrast, paging frequency increases as the day goes on, peaking towards the end of the shift. This is more pronounced at the University Hospital than at the VA, and more pronounced for interns than senior residents

Even within a given service, not all hours are created equal. Analysis of paging by year of trainee, clinical service, and hour of day sheds light on the cadence of various services (Fig. 2). For example, on call days, interns on the University inpatient medicine service are paged relatively infrequently during the morning, but paging begins to pick up around 1 p.m. and continues to increase all the way through the end of the admitting shift at 7 p.m. Pages to interns on the University night float service arrive in a bimodal distribution, peaking from 8 p.m. to midnight, and then again at 6 a.m., in the hour just before the shift ends, in what is known to many interns as the “morning labs” pages, when a new day’s worth of abnormalities must be acknowledged and acted upon.

### Relationship between paging burden and operational strain

To investigate whether variation in paging burden is actually informative of increased workload conditions, we selected the service with greatest volume of pages, the University night float service, for further study. This service also exhibited the greatest shift-to-shift variation in paging burden (Fig. 1). We then explored whether increased paging was associated a known metric of increased workload or operational strain.

Emergency department length of stay (ED LOS), and the related measure emergency department boarding time (the component of length of stay corresponding to time spent in the ED after the decision to admit has been made), are metrics known to increase under conditions of systemwide operational strain. Increased length of stay and boarding time are associated with adverse outcomes.^11,12^

**Fig. 3:**
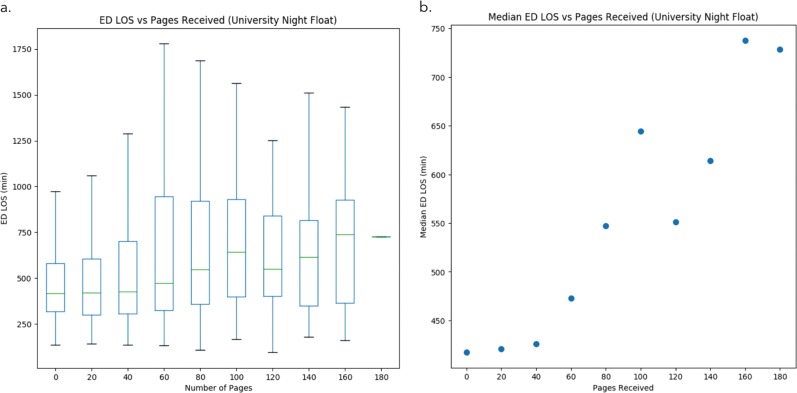
Relationship between ED length of stay for admitted patients and paging volume for a single service. **a** Emergency department length of stay (LOS), grouped by number of pages received, for nighttime admissions to University medicine admission and float service. While there exists variation, there appears to be a positively relationship between paging burden and LOS. **b** A zoomed-in view of median ED LOS vs. pages received more clearly depicts this relationship. The heaviest paging conditions correspond to observed median LOS more than five hours greater than observed median LOS under light paging. (boxplot elements: center line, median; box limits, upper and lower quartiles; whiskers, 1.5× interquartile range, outliers not shown)

A linear model was used to relate total emergency department length of stay and ED boarding time in minutes to number of pages received between the start of the shift and the time the medicine service was consulted for admission. Each additional page was associated with increased boarding time of 1.06 min per page (95% CI 0.51–1.32, *p*-value < 0.001), and increased overall length of stay of 1.16 min (95% CI 0.73–1.59, *p* < 0.001) (Fig. 3a). Light paging conditions were associated with median ED LOS of ~7 h, while the heaviest paging conditions correspond to observed median LOS of ~12 h in the period under study (Fig. 3b).

Time since shift start was not a significant factor, either independently or in combination with paging burden.

Nights with increased paging were independently, linearly, and statistically significantly associated with increased ED length of stay and boarding time, supporting the case for paging burden as a marker informative of increased operational workload.

## Discussion

Analysis of large-scale paging data—which we term PAGEOMICS (Paging Analytics Guided Exploration of Medicine in Clinical Settings)—can serve as a novel source of high-resolution information informative of resident workload and hospital operations. Paging burden can vary widely by service, hour of day, and year of training, even when hours worked are similar, and this variation is related to other systemwide measures of operational strain. This data can enable a more comprehensive understanding of not only duration, but also intensity of resident work. For example, on the University night-float service, each intern and resident works the same 12-hour shift nightly, from 7 p.m. to 7 a.m.; all house officers report essentially identical duty hours on this service. However, some house officers receive less than 10 pages on a shift while others receive over 80! In the quest to monitor resident workload and fatigue, it does not make sense to treat these two extremes as identical workloads. Paging data can provide information not be captured by duty hours reporting alone.

While this work establishes that paging data varies even for similar hours worked and explores the relationship between paging burden and operational strain, it does not characterize all the determinants of variation in paging burden. Future work can elucidate additional hospital factors (for example, patient census, team census, number of admissions and discharges), patient factors (for example, patient complexity or acuity), page factors (for example, content of page), and resident factors (for example, age, gender, prior performance) that may play a role in predicting paging burden. Similarly, other proxies for work intensity may be studied, whether metrics that can be obtained from the EHR, or external instruments such as survey data relating perception of shift intensity to paging burden.

Paging is an older technology, which may eventually be replaced by modern forms of communication, such as cell phones, texting, HIPAA-compliant messaging apps, and others. In any case, house officers will continue to receive inbound digital communications from various team members throughout the hospital, and studying the type and frequency of those communications is likely to provide insight into the nature and intensity of work undertaken by residents.

Paging data represents but one type of large-scale digital data generated over the course of daily hospital operation. The EHR provides another source—order-entry, keystroke logging, number of clicks, and time spent on documentation have all been observed or extracted for various studies.^13–15^ And even beyond hospital systems, wearable devices such as motion trackers, step counters, heart-rate monitors, and sleep trackers can potentially provide information on resident actions and, in some cases, physiological markers of stress and well-being. It is unlikely that any one data stream, whether paging or otherwise, will be universally informative for all residents on all services in all institutions. The approach outlined in this work—to first obtain the data and ensure sufficient data quality and quantity, then characterize baseline distributions and patterns of variation, and finally to evaluate whether that variation is informative of workload intensity in a particular context-outlines a framework for evaluating and incorporating novel data streams (or combinations of data streams) for workload monitoring. We refer to this approach as volume, variation, and validity (VVV).

The true opportunity with these novel class of digital data streams is not just in their richness, but in their timelines. Digital data streams, combined with real-time analytics and alerts, can finally move us from post-hoc reporting to proactive interventions. For example, automated reporting of an unusually busy shift may trigger an alert to a program director and prompt calling in a resident from a backup pool. Or a resident with several days in a row of high-workload conditions may be asked to take a day off for load-management, as is now done in some professional sports.^16^ Or perhaps persistently unequal distribution of workload intensity may induce redistribution of patients across teams. Rich, validated, real-time metrics of workload intensity make the study of these types of interventions possible, and provide an opportunity to not just reflect on adverse outcomes, but to avoid them.

A technical and policy framework to encourage exploration, development, evaluation, and use of these data-driven approaches is needed.

## Methods

### Data and analyses

The Stanford University Internal Medicine Residency Program accepts up to 50 interns each year; ~40 are categorical internal medicine residents. Residents perform inpatient rotations primarily at Stanford Hospital, a 613-bed tertiary care center, and the neighboring Palo Alto VA Hospital, which has 272 beds for general medical and surgical disciplines.

All paging IDs associated with residents active from July 1, 2013 to June 24, 2015 were identified. This period represents two complete academic cycles.

Metadata for all pages sent to these IDs during the study period, including the time the page was sent, was retrospectively obtained from the University’s office of Paging and Messaging Services. By cross-referencing with electronic call and service calendars and residency rosters, each page was computationally annotated with additional metadata including the year of training of the recipient, the clinical service, and for inpatient services, whether the recipient was on call. Text of alphanumeric pages was not available for this study.

Emergency department length of stay and boarding time data for all general medicine admissions at the University hospital during the study period was obtained.^17^ Of the 9516 admissions to the service during the study period, we included only admissions to the night float service, and only those which had nonmissing, chronologically ordered timestamps for ED arrival, ED “Consult to Medicine” order, “Admit to Medicine” order, and ED departure. Two thousand three hundred and twenty five admissions (24.4%) met this inclusion criteria. A linear model was used to relate emergency department length of stay and boarding time in minutes to number of pages received between the start of the shift and the time the medicine service was consulted for admission.

This study was approved by the Stanford University Institutional Review Board (protocol #42093) with waived informed consent due to the retrospective nature of the study.

### Reporting summary

Further information on research design is available in the Nature Research Reporting Summary linked to this article.

## Supplementary information

Checklist-Reporting Summary

## Data Availability

Data are available from the corresponding author (A.K.). The data are not publicly available due to them containing information that could compromise research participant privacy or consent, as well as patient privacy or consent.

## Supplementary information

**Supplementary information** accompanies the paper on the *npj Digital Medicine* website (10.1038/s41746-019-0165-2).
